# Distinct Profiles of 50 kHz Vocalizations Differentiate Between Social Versus Non-social Reward Approach and Consumption

**DOI:** 10.3389/fnbeh.2021.693698

**Published:** 2021-06-21

**Authors:** Mohammad Seidisarouei, Sander van Gurp, Nicole Melisa Pranic, Irina Noguer Calabus, Marijn van Wingerden, Tobias Kalenscher

**Affiliations:** ^1^Social Rodent Lab, Institute of Experimental Psychology, Heinrich-Heine-University, Düsseldorf, Germany; ^2^Comparative Psychology, Institute of Experimental Psychology, Heinrich-Heine-University, Düsseldorf, Germany; ^3^Department of Psychology, Cornell University, Ithaca, NY, United States; ^4^Department of Cognitive Science and Artificial Intelligence, Tilburg School of Humanities and Digital Sciences, Tilburg University, Tilburg, Netherlands

**Keywords:** ultrasonic vocalizations, social, behavior, reward processing, rats, vocal communications, 50 kHz calls, subtypes

## Abstract

Social animals tend to possess an elaborate vocal communication repertoire, and rats are no exception. Rats utilize ultrasonic vocalizations (USVs) to communicate information about a wide range of socially relevant cues, as well as information regarding the valence of the behavior and/or surrounding environment. Both quantitative and qualitative acoustic properties of these USVs are thought to communicate context-specific information to conspecifics. Rat USVs have been broadly categorized into 22 and 50 kHz call categories, which can be further classified into subtypes based on their sonographic features. Recent research indicates that the 50 kHz calls and their various subtype profiles may be related to the processing of social and non-social rewards. However, only a handful of studies have investigated USV elicitation in the context of both social and non-social rewards. Here, we employ a novel behavioral paradigm, the social-sucrose preference test, that allowed us to measure rats’ vocal responses to both non-social (i.e., 2, 5, and 10% sucrose) and social reward (interact with a Juvenile rat), presented concurrently. We analyzed adult male Long-Evans rats’ vocal responses toward social and non-social rewards, with a specific focus on 50 kHz calls and their 14 subtypes. We demonstrate that rats’ preference and their vocal responses toward a social reward were both influenced by the concentration of the non-social reward in the maze. In other words, rats showed a trade-off between time spent with non-social or social stimuli along with increasing concentrations of sucrose, and also, we found a clear difference in the emission of flat and frequency-modulated calls in the social and non-social reward zones. Furthermore, we report that the proportion of individual subtypes of 50 kHz calls, as well as the total USV counts, showed variation across different types of rewards as well. Our findings provide a thorough overview of rat vocal responses toward non-social and social rewards and are a clear depiction of the variability in the rat vocalization repertoire, establishing the role of call subtypes as key players driving context-specific vocal responses of rats.

## Introduction

Rats are social animals (Whishaw and Kolb, 2009) that form relatively large and tightly organized groups. As nocturnal animals, many rodent species rely on complex vocalizations for communication and social coordination. The extent of their vocalization vocabulary depends on their social structure and inter-individual interactions (for a review, see Brudzynski, 2014). Among rodents, rats, in particular, have developed an elaborate system of ultrasonic communication which has been suggested to have adaptive significance by signaling socially relevant information: ultrasonic vocalizations (USVs) emitted by rats have been implied to play a role in warning conspecifics (Litvin et al., 2007; Brudzynski, 2013), as well as acting as indices of rats’ affective states (Knutson et al., 2002; Brudzynski, 2013) and social motivation (Mulvihill and Brudzynski, 2018b). Additionally, Himmler et al. (2014) have demonstrated the function of rat USVs in facilitating and maintaining play behavior, pointing to their social communicative value. Thus, it has been suggested that the wide range of calls emitted by rats serve a multitude of context-dependent functions.

The USVs emitted by pups, adolescent and adult rats can be divided to three major sub-groups: (i) 22 kHz alarm calls (Litvin et al., 2007) produced in response to an aversive circumstance (Wöhr and Schwarting, 2013), (ii) 50-kHz USVs that signal appetitive and rewarding states (Panksepp and Burgdorf, 2000) and (iii) 40 kHz vocalizations produced by socially isolated pups (Wöhr et al., 2008). The acoustic features of the 50 kHz calls differ substantially from 22 kHz USVs (Brudzynski and Pniak, 2002; Brudzynski and Holland, 2005; Thompson et al., 2006), allowing distinct and clear-cut classifications. Specifically, 50 kHz USVs have a concise call duration between 30 and 40 ms, a bandwidth of 5–7 kHz, and a peak frequency remaining within 45–55 kHz, although the calls can reach 70 kHz or higher.

The 22 and 50 kHz call categories emitted by rats thus represent general qualitative information regarding the condition of the environment or behavior, but these call categories can be further organized into subtypes of vocalizations (Wright et al., 2010; Himmler et al., 2014; Brudzynski, 2015) that differ in sonographic features. For instance, 50 kHz USVs can be classified into Flat and frequency-modulated (FM) subtypes based on the bandwidth of frequencies they extend over in spectrograms (Burgdorf and Panksepp, 2006; Wöhr et al., 2008). Several lines of evidence demonstrate that rats emit Flat- and FM-50 kHz USVs in different situations, suggesting that these subgroups of 50 kHz USVs may have distinct and disparate communicative roles of behavioral significance. Flat calls, for instance, have been suggested to be involved in (initiating) social contact (Burgdorf et al., 2011) and social coordination (Wöhr and Schwarting, 2008). FM 50 kHz USVs, on the other hand, are more commonly emitted during rewarding situations or high positive emotional arousal (Burgdorf et al., 2011). The FM subgroup of 50 kHz USVs have been further grouped into subtypes based on the extent of their frequency modulation and the shape they assume in the spectrogram (Brudzynski and Zeskind, 2018). In the most comprehensive classification, the 50 kHz USVs were categorized into 14 distinct subtypes (Wright et al., 2010). This categorization, however, is not one without controversy. Coffey et al. (2019), for instance, have recently utilized the DeepSqueak software to classify USVs using unsupervised machine learning techniques into 18 separate clusters instead of 14 subtypes. In addition, the behavioral relevance of these various call subtypes remains largely unknown.

Because of their association with appetitive situations, 50 kHz calls could potentially also be utilized in quantifying the value that individual rats attribute to a reward (Garcia et al., 2015) as well as to the expectation of a reward (Binkley et al., 2014). Calls emitted in the presence of non-social and social rewards have been investigated thoroughly in the literature. Cues for nutritional reward have been shown to elicit 50 kHz responses from rats (Brenes and Schwarting, 2014), and a preference for sweet pellets over regular pellets is associated with an increase in the frequency of 50 kHz vocalizations (Mateus-Pinheiro et al., 2014). Nevertheless, Schwarting et al. (2007) found no difference between the 50 kHz calls produced by food-deprived animals and the ones exposed to ad-libitum feeding, when they were alone in the home cage. In another intricate design, Browning et al. (2011) have demonstrated that rats trained for cocaine and sucrose self-administration showed more 50 kHz calls during the reward self-administration and reinstatement phase (after a period of extinction training), compared to naïve controls who were not rewarded.

Juvenile, adolescent, and adult rats have been shown to emit 50 kHz calls during interactions with their conspecifics, such as rough and tumble play (Knutson et al., 1998) and mating (White et al., 1990). Female rats also produce 50 kHz calls when encountering a social partner (Börner et al., 2016). The calls emitted by adult rats can thus give clues about their social behavior (but see, Manduca et al., 2014). It has been shown that rats emit more 50 kHz calls when exposed to another conspecific (Brudzynski and Pniak, 2002) and display a preference for rats producing more 50 kHz calls (Panksepp et al., 2002). In contrast, rats selectively bred to emit lower rates of 50 kHz calls spent less time with conspecifics in a social interaction test than the randomly bred line (Burgdorf et al., 2009). Similarly, playful experiences are significantly less frequent in pairs of devocalized rats than in their vocalizing counterparts, emphasizing the role of these 50 kHz calls in maintaining play behavior (Himmler et al., 2014).

Łopuch and Popik (2011), Kalenscher (2020), and Kalenscher et al. (2021) have also argued that the cooperative behavior of rats positively correlates with the 50 kHz vocalizations they produce, as 50 kHz USVs may act as social vicarious reward signals (Hernandez-Lallement et al., 2016; Van Gurp et al., 2020; Löbner et al., 2021). Neural processing of USVs has been implicated in the amygdala, with opposing coding schemes for 22 vs. 50 kHz USVs (Parsana et al., 2012), and indeed, lesions of the BLA impair the social approach that is usually observed to 50 kHz USV playback (Wöhr and Schwarting, 2007; Seffer et al., 2014; Schönfeld et al., 2020).

In short, both qualitative and quantitative differences in 50 kHz USV production have been found across a range of social and non-social rewarding situations. Only a handful of studies in the literature, however, have investigated USV production in the context of concurrent social and non-social rewards. Utilizing selective breeding procedures (Burgdorf et al., 2009), have demonstrated that rats bred to emit higher rates of 50 kHz calls were more likely to prefer a sucrose solution to tap water than randomly bred rats. Willey and Spear (2013) analyzed the calls and approach behavior toward both food-related and social stimuli in rats exposed to varying degrees of social deprivation. The time animals spent investigating the social stimulus within the apparatus positively correlated with the frequency-modulated (FM) calls they emitted. However, these authors did not find a relationship between animals’ responses to food stimuli and their USV production. In a novel design, Mulvihill and Brudzynski (2018b) analyzed the USVs produced by male rats separately allowed to freely explore a female, a littermate, as well as two non-social conditions, namely Fruit Loop rewards and 2% ethanol solution. Their results indicated that out of the four groups, only rats exposed to a cycling female produced a higher proportion of calls than the baseline. Mulvihill and Brudzynski (2018b) also demonstrate significant differences between the types of calls made in non-social versus social conditions. Specifically, rats exposed to non-social stimuli produced more flat calls than non-trill FM calls, whereas the non-trill FM subtype dominated the 50 kHz calls in the social contexts.

Thus, in summary, there is growing evidence that 50 kHz USVs, and the 50 kHz subtypes, are related to the subjective experience of social vs. non-social rewards, which could be related to reward processing traits (such as sucrose preferences), to individual communicative traits, or a combination of these factors. If there indeed is a structure to the type of vocalizations emitted in social and non-social situations, akin to a selective “vocabulary” for different behavioral contexts, it should be possible to distinguish these contexts when presented in direct competition, based on the vocalization patterns that are recorded.

To study this question, we employed a novel behavioral paradigm, the social-sucrose preference test. It is conducted on an XCST (X-shape chambered sociability test) maze. The XCST maze is a modified version of a radial arm maze previously utilized by Schönfeld et al. (2020) that can be used to contrast behavioral responses to both a social reward (Juvenile conspecific in an open-bar sociability cage) and varying levels of non-social reward (sucrose solutions) in different arms of the apparatus while recording the USVs emitted by the animals. Thus, we systematically investigated how the occurrence of the 14 subtypes of rat USVs was related to rats’ choice behavior in the trade-off between social and non-social rewards.

## Materials and Methods

### Subjects

The experiment was conducted according to the European Union Directive 2010/63/EU for animal experimentation and was approved by the local authority (Landesamt für Natur, Umwelt und Verbraucherschutz North-Rhine Westphalia, Germany). Fifteen male Long-Evans rats (*Charles River, Italy*) in total were obtained in a batch of 12 experimental animals (PND 40, Mw*_*eight*_* = 320 g, at the starting day of the experiment) and 3 Juvenile rats [PND 28, *M*_*weight*_ = 290 g, at the starting day of the *Social-Sucrose Preference Test (SSPT)*], serving as social stimulus/reward. Experimental rats were housed in groups of *N* = 3 rats in standard Type IV Macrolon cages under a reversed 12:12 h light-dark cycle. The housing room was kept at a constant temperature of 22°C and a humidity of 60%. Throughout the experiment, all rats received standard laboratory rodent food, *ad libitum*, except for the Sucrose Discrimination Test (SDT) phase in which all experimental animals were limited in their food intake (food per rat per day: 22 g on weekdays and 25 g on weekends).

### Behavioral Task Setup

**FIGURE 1:**
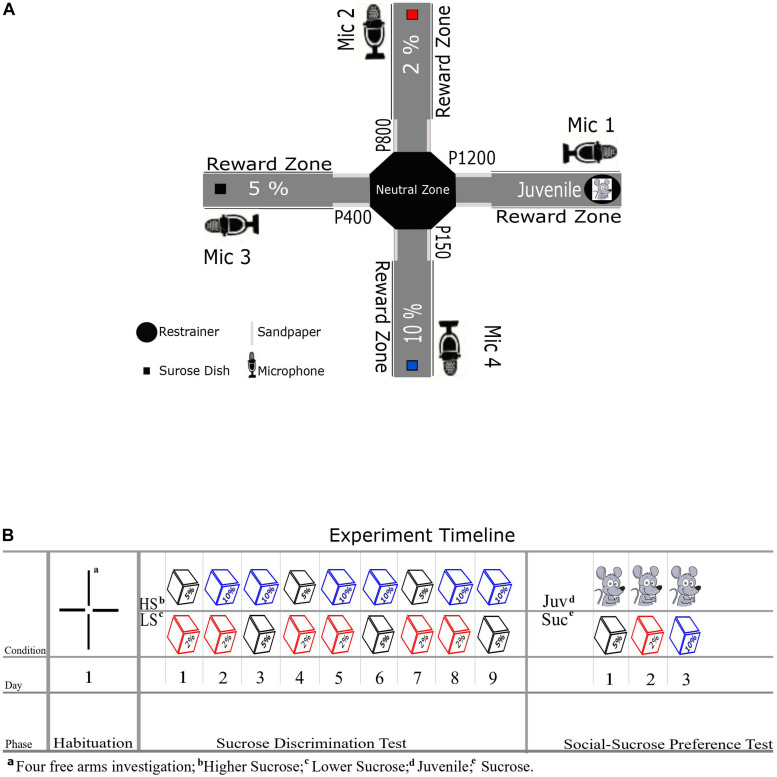
**(A)** Schematic diagram of XCST setup with non-social reward positions, the restrainer for the juvenile social reward, sandpaper positions, and microphones. Every arm was assigned to a specific reward throughout the experiment. **(B)** Shows the experiment timeline for different phases, days, and conditions—the cubes represent the sucrose in different concentrations.

We used an eight-arm radial maze as previously adapted by Schönfeld et al. (2020), detached four arms to arrive at a cross/plus-maze setup (Figure 1A). The maze consisted of a central platform (36 cm diameter; so-called neutral zone in our design) and four arms (14 cm wide and 60 cm long) that extended from the central platform in an octagon platform. Each of the four arms was consistently associated with one single reward type: 3 arms with three different levels of a sucrose solution reward (see Figure 1A) and one arm with a social stimulus. To circumvent any spatial bias, we divided our subjects into two groups (A and B, per group = 6) with a different allocation of reward positions for each group. Notably, during any test day in the experiment, only 2 out of 4 arms were open at a time to provide a head-to-head preference test between two rewards. On the arm of the maze assigned to the social reward, an unfamiliar Juvenile rat could be placed in a fixed cylindrical restrainer built from metal bars and compact plastic for its floor and ceiling (Height: 25.5 cm, Diameter: 17 cm, Ugo Basile Sociability Cage). The restrainer was fixed on the maze at the end of the Juvenile’s arm, and the Juvenile could move around in this restrainer, and social contact through the openings between the bars was possible. On the arms allocated to non-social reward (i.e., different sucrose concentrations 2, 5, and 10%), sucrose solution was provided to the experimental animal in a cube plastic dish (8 × 8 cm) mounted at the end of each arm. Additionally, in order to facilitate spatial learning of the reward conditions in each arm over days, we included sandpapers (17 × 13 cm) in the entrance of each arm that the rats’ whiskers touch when entering the arms. The sandpapers had varying grades (Group A:2% [P800], 5% [P400], 10% [P150], and Juvenile [P1200], Group B: 2% [P150], 5% [P1200], 10% [P800], and Juvenile [P400]), following the findings of Guić-Robles et al. (1989) These authors have demonstrated that rats’ whiskers can discriminate between sandpapers with 200 and 25 grains/cm^2^. To record the ultrasonic vocalizations (USVs), four ultrasonic microphones (Condenser Microphone CM16/CMPA, Avisoft Bioacoustics, Glienecke, Germany) were positioned via a microphone stand to approximately 20 cm on the right side of each reward dish and the restrainer (see Figure 1A).

### Social-Sucrose Preference Test Design (SSPT)

**FIGURE 2:**
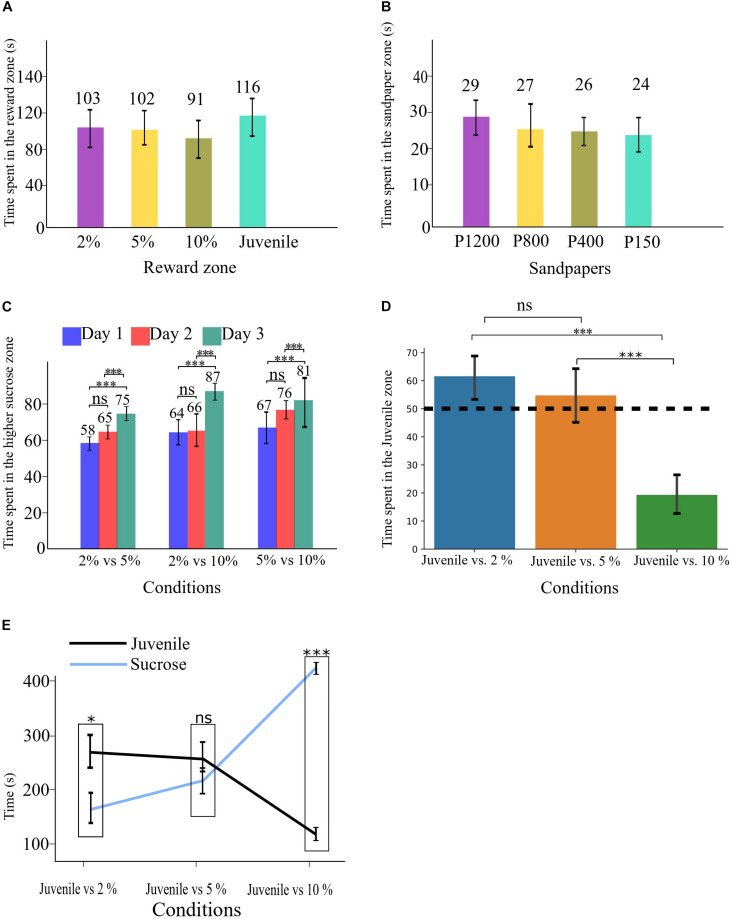
**(A)** Time spent in each reward zone during the Habituation phase. **(B)** Time spent in each sandpaper zone during the Habituation phase. **(C)** Time spent in the higher sucrose zone in all three conditions of SDT. **(D)** time spent in the Juvenile zone in all three conditions of SSPT, the dashed line shows the 50% point. **(E)** Absolute time spent in each reward zone for all three conditions. All error bars show the standard deviation. ^∗^*p* < 0.05, ^∗∗∗^*p* < 0.001.

Behavioral testing on the SSPT included three phases (see Figure 1B). In all phases of this study, experimental animals started the trials from the neutral zone facing not toward targeted arms in given condition. In the first habituation phase, all four arms were open and unbaited, and each experimental animal explored the maze for 10 min. This phase aimed to find out whether animals were inherently biased toward selecting one specific reward zone or sandpaper (see Figures 2A,B). The second phase of training was the Sucrose Discrimination Test (SDT), which was implemented to verify that the experimental animals could indeed distinguish among the three selected sucrose concentrations (2, 5, and 10%). Food deprived animals were tested on the SDT phase over 9 days in three repetitions of three different conditions. In each condition, only two arms were open, and rats chose to allocate their time between rewards on the maze in the following order of conditions: *2% vs. 5%*, *2% vs. 10%*, and *5% vs. 10%*. Notably, each animal was tested in only one condition each day. Each test trial took 10 min; during this time, experimental animals could move freely in the two open arms and drink up to 20 ml sucrose solution per plastic dish at the end of each arm. Both dishes were filled with fresh sucrose solution for each new trial/experimental animal. After passing the SDT phase (Figure 2C), the experiment was continued to the SSPT phase. In this phase, over each trial with a duration of 10 min, the experimental animal could similarly move freely between two open arms: either to explore the arm baited with sucrose, or to investigate the Juvenile rat in the restrainer at the end of the Juvenile arm. Animals were tested once per day in three conditions (Juvenile vs. 2%, Juvenile vs. 5%, and Juvenile vs. 10%) spread out over the three SSPT testing days (see Figure 1B). To keep baseline motivation equal for both types of reward (social vs. non-social), food deprivation was stopped after the final SDT test day, and animals were allowed to recover weight over 2 days before starting the SSPT. For the remainder of the experiment, animals were kept *ad libitum*. Rats usually spend more time exploring novel conspecifics than familiar ones (Smith et al., 2015, 2017), suggesting that the value of social interaction dynamically decreases over days with increasing familiarity with the conspecific. To keep the novelty, and, hence, the value of investigation of the social stimulus similar across testing sessions, three different Juvenile rats were used in all three conditions of SSPT for each experimental animal. The order of the identities of these Juveniles was counterbalanced across experimental animals to exclude identity effects. All USVs from all trials over the two phases (SDT and SSPT) were recorded for the full 10-min trial duration, with the sampling rate set at 250 kHz.

### Behavioral Analysis: Video-Tracking

For the recorded videos from all sessions, Ethovision (EthoVision XT version 11.5, Noldus) was used to track the animals’ position. Tracking settings were optimized separately for each different phase of the study (Habituation, SDT, SSPT). In the habituation phase, each arm was divided into two zones (Sandpaper zone and Reward zone) to check for any inherent bias for the different reward zones and sandpaper zones. For the SDT and SSPT phases, we used the time that the animals spent in the reward zones (see reward zones; Figure 1A). The time spent in the neutral zone was excluded from the analysis.

### Ultrasonic Vocalization Recording, Labeling Procedure, and Synchronization

Acoustic analysis of the USVs was executed using the software Avisoft-SASLab Pro (Version 5.2, Avisoft Bioacoustics, Berlin, Germany). Spectrograms were generated with a fast Fourier transform (FFT)-length of 512 points and an overlap of 75% (Flat Top window, 100% frame size). Correspondingly, spectrograms had a frequency resolution of 390 Hz and a time resolution of 0.64 ms. In the setup, we recorded the USVs through 4 microphones, providing a four-channel spectrogram recording. The amplitude of the USVs differed depending on the distance between the animal and the different microphones (Supplementary Figure A). The microphone channel that recorded the largest amplitude was selected for labeling for each USV in the spectrograms. This channel differed between the conditions and minutes of the trial. The labeling phase was conducted by two trained, independent scorers who labeled and classified each USV based on its sonographic features (as in Wright et al., 2010). Notably, in the SSPT phases, calls could be emitted by both the experimental animal and the Juvenile social stimulus. In these analyses, we did not attempt to tease apart the source of these vocalizations but instead rely on within-subject comparisons of experimental animals to quantify differences.

**FIGURE 3:**
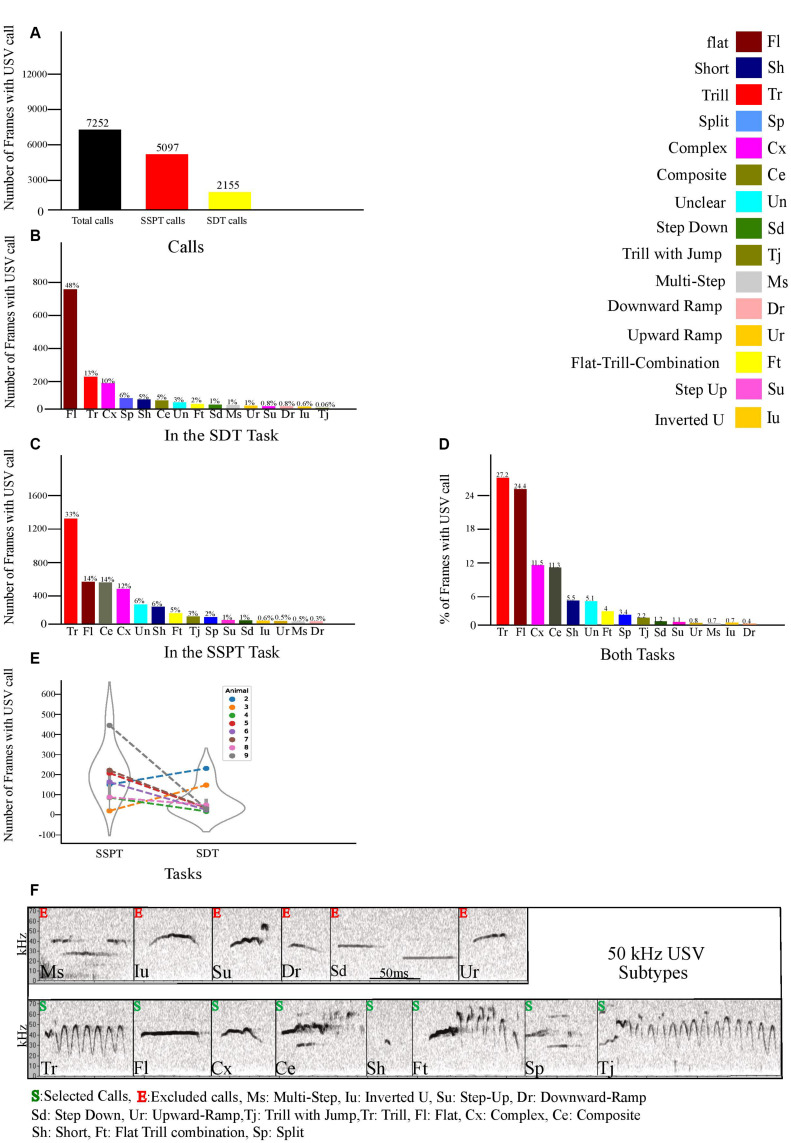
**(A)** Number of frames animals vocalized in both tasks and separately during each task. **(B)** Number of call frames for each distinct 50 kHz subtype in the SDT task. **(C)** Number of call frames of each 50 kHz subtype in the SSPT. **(D)** Percentage of each subtype vocalized in both tasks. **(E)** Number of call frames per animal per task averaged over all three conditions. **(F)** Examples of the fourteen 50 kHz USV Subtypes (labeled according to Wright et al., 2010). Subtypes are marked with S (selected) or E (excluded, see text).

The labeling phase consisted of two steps: calibration and final labeling. During the first step, two scorers became familiar (under the supervision of the expert scorers) with sonographic features of each of the 50 kHz USV subtypes (and 22 KHz) according to the classification suggested by Wright et al.(2010; for an overview of the different USV subtypes considered in this study, Figure 3F). They initially labeled USVs together to reach a consensus labeling scheme. After this calibration step, they separately labeled the same 400 USVs and, subsequently, compared their labeling match. In total, inter-rater reliability was high (Cohen’s kappa = 0.95), such that 94.3% of 50 kHz USV’s subtypes were labeled with the same category by both scorers. Due to technical problems, the USV files of the condition *2% vs. 5%* and some animals (1,10,11,12) from the SSPT task were lost. Therefore, for all USV related statistical analyses, we only applied the USVs from 8 animals for both tasks. Thirty-two trials from SDTs’ phase, including 2 days (2 and 3) for conditions (*2% vs. 10%* and *5% vs. 10%*), were labeled. For the USVs from the SSPT phase, the recordings from all three test days (*N* = 24 recordings in total) were labeled. Both scorers tagged half of all USVs from the same conditions (every odd minute of each trial).

### USV Call Production Definition and Behavior-USV Synchronization

When labels were assigned in Avisoft, through the self-written code in python, we exported the USV raw data (Avisoft SAS-Lab Pro’s output) to generate a time series of vocalization labels with a temporal resolution of 25 Hz, synchronized to the video stream and position data (Ethovision output). Thus, each 0.040 ms sample had a one-hot encoded binary label, corresponding to the presence/absence of each of the 50 kHz subtypes, 22 kHz or background/noise. We first looked at the summed frames spent vocalizing, including all rats, to establish inclusion/exclusion criteria. The 22-kHz USVs accounted for 23.3% of all samples with USVs, counted in ms spent vocalizing. This high proportion of 22 kHz frames is mainly caused by the naturally longer length of a 22 kHz USV compared to the length of a 50 kHz call. As the main goal of this experiment only covers the 50 kHz calls, no further analysis was conducted on the 22 kHz calls. Figure 3E shows the inter-individual variation in USV production, warranting a within-subjects approach that includes normalization to correct these inter-individual differences in calculating group contrasts (see below). During the labeling phase, 3.9% of all call frames could not be clearly labeled in any of the 14 categories of 50 kHz subtypes. These USVs with varying sonographic features were called Unclear (Un, and Supplementary Figure B) and excluded from USVs within-between analyses. After labeling all 50 kHz USVs, six subtypes (Step-Down, Inverted-U, Step-Up, Multi-Step, Downward Ramp, and Upward Ramp) were excluded because of their small incidence (<2% of all call frames [an arbitrary cut-off]). The selected call subtypes were thus: Trill, Flat, Complex, Composite, short, Flat-Trill-combination, Split, and Trill-with-Jump.

### Statistical Analyses

#### Behavioral Analyses

To rule out any spatial biases for or against some arms over others in the maze, independent of the reward contingencies, we applied independent samples *t*-tests to check for differences in time spent in each reward zone between groups A and B. To check for spatial bias related to any inherent preference for the different reward zones and sandpapers, we performed a repeated-measures ANOVA to assess the effect of sandpaper type and reward zones as independent variables (IVs) on the time animals spent in each reward and sandpaper zone during habituation to the maze, when rewards were not yet introduced. To find out whether rats discriminated between different sucrose levels in the SDT, first, we calculated the SDT sucrose solution preference score for each day/condition in the SDT as a percentage of time spent with the higher sucrose (Figure 2C).

$$\mathrm{SDT}\,\text{sucrose}\,\mathrm{preference}\,\mathrm{score}=\frac{\begin{array}{c} \mathrm{Time}\,\mathrm{spent}\,\mathrm{in}\,\mathrm{high}\\ \mathrm{sucrose}\,\mathrm{reward}\,\mathrm{zone} \end{array}}{\begin{array}{c} (\mathrm{Time}\mathrm{spent}\mathrm{in}\mathrm{high}\mathrm{sucrose}\mathrm{reward}\mathrm{zone}+\\ \mathrm{Time}\,\mathrm{spent}\,\mathrm{in}\,\mathrm{low}\,\mathrm{sucrose}\,\mathrm{reward}\,\mathrm{zone} \end{array})}*100$$

with these sucrose preference scores, we conducted a two-way repeated-measures ANOVA with the condition (three levels: *2% vs. 5%*, *2% vs. 10%*, and *5% vs. 10%*) and task repetition day (three levels: days 1, 2, 3) as (IVs) and % time spent in the higher sucrose zone as dependent variable (DV) (Figure 2D).

Similarly, for the SSPT task, first, we calculated a Juvenile preference score,

$$S\,S\,P\,T\,\mathrm{Juvenile}\,\mathrm{preference}\,\mathrm{score}=\frac{\begin{array}{c} \mathrm{Time}\,\mathrm{spent}\,\mathrm{in}\,\mathrm{the}\\ \mathrm{Social}\,\mathrm{Reward}\,\mathrm{zone} \end{array}}{\begin{array}{c} (\mathrm{Time}\mathrm{spent}\mathrm{in}\mathrm{the}\mathrm{Social}\mathrm{Reward}\mathrm{zone}+\\ \mathrm{Time}\,\mathrm{spent}\,\mathrm{in}\,\mathrm{the}\,\mathrm{Non}-\\ \mathrm{social}\,\mathrm{reward}\,\mathrm{zones} \end{array})}*100$$

and used this Juvenile preference score to run a repeated-measures ANOVA to detect any differences in Juvenile preference as a function of sucrose concentration (*Juvenile vs. 2%*, *Juvenile vs. 5%*, and *Juvenile vs. 10%*). To find out if animals preferred a particular reward type over the other in each condition, we analyzed their preference by applying a paired samples *t*-test. Finally, regarding the design of the maze, animals could also spend their time in the Neutral zone, as SSPT Juvenile preference score only considered the percentage of the time animals spent in reward zones, in order to know whether animals spent different time for a particular reward (either Social or Non-social) over the three conditions, we conducted a two-way repeated-measures ANOVAs with Conditions and Zone as IVs and absolute time spent per reward zone as DV, and performed *post hoc* paired-sample *t*-tests to compare the absolute time spent between zones per condition. For all statistical analyses, the significance level was *p* < 0.05, and all the *post hoc* tests *p*-values were Bonferroni-corrected for multiple comparisons.

### Vocalization Analyses

Our initial analysis focused on a Combined vocalization score (CVS), including all 15 subtypes (Including Un and excluding only the 22 kHz) per session to look for overall differences in vocalization rates between conditions. Here, we first summed up all frames the rats vocalized for each of the 15 subtypes in a certain zone and then divided that score by the time the animal spent in that zone, thus normalizing the vocalization time to the occupation time per zone, creating a normalized vocalization rate. As inter-individual differences resulted in a skewed distribution of normalized vocalization rates, we performed a log transformation on these CVSs to reduced skewness and facilitate visualization. To investigate if the number of vocalizations differed depending on the reward type (social vs. non-social) or sucrose concentration, we applied a two-way repeated-measures ANOVA for each task (SDT and SSPT) separately. Here, we considered the condition with two levels for SDT (conditions: 2 vs. 10% and 5 vs. 10%), three levels for SSPT (*Juvenile vs. 2%*, *Juvenile vs. 5%*, and *Juvenile vs. 10%*), and two levels reward zone (SDT: higher/lower sucrose and SSPT: Juvenile/Sucrose) as IVs, and the log of the CVS of each task as DV.

To zoom in to differences between subtypes, we performed a similar analysis pipeline per subtype: after excluding the 22 kHz, Un, and infrequent call subtypes (excluded calls), for the remaining eight categories, we again normalized the subtype-specific vocalization rate to the spatial occupancy per zone to calculate a subtype vocalization score (SVS). This SVS was thus calculated by summing the number of frames the rat vocalized a specific subtype (1 frame = 0.040 ms) in a given zone and dividing it by the time the animal spent in that zone.

As a within-subjects normalization step, from these SVSs, we calculated a delta SVS score to show the differences in vocalization rate between zones for a given subtype. The delta SVS score was calculated as follows: i) SVS score in the low sucrose zone subtracted from the SVS score in the high sucrose zone for SDT and ii) SVS score in the non-social reward zone subtracted from the SVS score in the social reward zone for SSPT. We used this deltaSVS to compare normalized vocalization rates between subtypes in a given condition (between-subtype analyses) and within a subtype, between conditions (within-subtype analyses).

In the between-subtype analysis, with these dSVS, we ran a Kruskal Wallis test per condition for the SDT and SSPT data, with the subtype as the IV and the dSVS score as DV for each condition.

In the within-subtype analysis, we performed a Wilcoxon Signed-Rank test for the SDT sessions, comparing the vocalization of the given subtype in two conditions [*2% vs. 5%* and *5% vs. 10%*]) and a Friedman test for each subtype across the three SSPT conditions (*Juvenile vs. 2%*, *Juvenile vs. 5%* and *Juvenile vs. 10%*). For all statistical analyses, the significance level was set at *p* < 0.05, and all the *post hoc* tests *p*-values are Bonferroni- corrected for multiple comparisons.

### Mixed Linear Model Analyses

To exploit the continuous range of sucrose solutions used in the SSPT, to look for a linear association between vocalizations and sucrose solution, we conducted two mixed linear models, one on total calls (CVS) and one on subtype-specific SVS. Both models entered Animals as random effects, Conditions (2% vs. Juvenile, 5% vs. Juvenile, and 10% vs. Juvenile) as fixed effects, and CVS/dSVS as the dependent variable.

### Software

All statistical analyses were carried out using SPSS Statistics (version 24; IBM, United States) and R 3.5.1 (R Core Team, 2018). We applied the following libraries in R: the tidyverse (Wickham, 2017), the haven psycho, the readxl (Wickham et al., 2019b), the tidyr (Wickham et al., 2019a), the tibble (Wickham et al., 2019a), the sjplot (Lüdecke, 2020) the ggstatsplot (Patil, 2018) and the rockchalk (Johnson and Grothendieck, 2019). Moreover, visualizations of some figures (Figure 2D and Supplementary Figure D) were made using Jupyter Notebook (Kluyver et al., 2016) through the packages matplotlib (Hunter, 2007), pandas (McKinney, 2010), and seaborn (Waskom, 2021). Remaining figures were created by Inkscape (version 0.92.1, Inkscape project, 2020). In order to run the synchronization of USV and Animals’ positions we used the packages fileinput (Sinha, 2017) numpy (Harris et al., 2020).

## Results

### Behavior

A between-group comparison did not find evidence for a difference in spatial/reward preference based on the maze layout for groups A and B (Supplementary Figures CA,CB). Similarly, an analysis of the habituation period did not find any evidence for a preference for a specific zone of reward [*F*(3, 33) = 1.35, *p* > 0.05; Figure 2A] or sandpaper zone [*F*(3, 33) = 1.6, *p* > 0.05; Figure 2B].

**SDT**. To determine whether experimental animals could indeed discriminate between different sucrose concentrations (i.e., 2, 5, and 10%), we conducted a two-way repeated-measures ANOVA with task condition and task repetition day as within-subject factors and percentage of the higher sucrose reward as DV. We found no significant main effect of task condition, suggesting that animals did not significantly differ in their preference for the sweeter sucrose solution across sessions with different levels of sucrose concentrations. We did observe a significant main effect of day [*F*(2, 22) = 15.2, *p* < 0.001, η*_*p*_*^2^ = 0.581]. *Post hoc* analysis revealed that animals preferred the higher-percentage sucrose solution significantly more in all conditions on day three (*M* = 81.7, *SE* = 2.8) compared to day two (*M* = 69.1, *SE* = 2.7, *p* < 0.05, *d* = 4.6) and day one (*M* = 63.3, *SE* = 2.7, *p* < 0.001, *d* = 6.8). The data thus showed that animals develop a clearer preference for the sweeter sucrose solution over days (Figure 2C), probably as a consequence of learning. There was no significant interaction effect.

**SSPT**. To assess whether animals expressed a significant preference between social and non-social rewards (with three different sucrose concentrations) in the social-sucrose preference test (SSPT), we conducted a one-way repeated-measures ANOVA on the percentage of time spent with the social reward (Juvenile zone). The results showed that preferences for the Juvenile differed significantly between conditions [*F*(2, 22) = 52.2, *p* < 0.001, η*_*p*_*^2^ = 0.826]. *Post hoc* tests revealed that the animals’ preference for the Juvenile increased significantly from the condition *Juvenile vs. 10%* (juv. pref: *M* = 19%, *SD* = 10%) condition to the *Juvenile vs. 5%* (juv. pref: *M* = 55%, *SD* = 15%, *p* < 0.001, *d* = 12.2) condition. There was a further but non-significant increase in Juvenile preference when reducing the sucrose concentration to 2%; in this condition, Juvenile preference was also significantly higher than in the *Juvenile vs. 10%* condition (juv. pref: *M* = 61%, *SD* = 13%, *p* < 0.001, *d* = 9.4). Three one-sample *t*-tests vs. indifference (50%) showed that animals preferred the social reward in Juvenile vs. 2% [*M* = 61.5, *SD* = 13, *t*(11) = 3.06, *p* < 0.05], were indifferent between Juvenile vs. 5% [*M* = 54.7, *SD* = 15, *t*(11) = 1.08, *p* > 0.05] and preferred the sucrose reward in Juvenile vs. 10% [*M* = 80.6, *SD* = 10, *t*(11) = 9.9, *p* < 0.001]. These results show clearly that animals indeed traded off interacting with a Juvenile to the consumption of sucrose and also that a preference for interacting with the Juvenile when sucrose levels were low (2%) could be reversed when confronted with a more preferred 10% sucrose solution (Figure 2D). These between-condition differences could be due to a change in time (%) spent at the sucrose reward, the social reward, or both. To quantify this, we investigated if the absolute time animals spent in each reward zone differed between different conditions. A repeated-measures ANOVA on the absolute time animals spent on social reward showed a significant effect of conditions [*F*(2, 22) = 33.2, *p* < 0.001, η*_*p*_*^2^ = 0.751]. *Post hoc* tests revealed that the absolute time that animals spent in the Juvenile zone in the condition of *Juvenile vs. 10%* (*M* = 97.7, SD = 55) was significantly less than in the condition *Juvenile vs. 5%* (*M* = 250, *SD* = 76, *p* < 0.001, *d* = 2.2) and the condition *Juvenile vs. 2%* (*M* = 259, *SD* = 64, *p* < 0.001, *d* = 2.7). There was no significant difference between the condition *Juvenile vs. 2%* and *Juvenile vs. 5%*. A second repeated-measures ANOVA on the absolute time animals spent with non-social rewards also showed a significant effect of the condition [*F*(2, 22) = 74.7, *p* < 0.001, η*_*p*_*^2^ = 0.872]. Here, *post hoc* tests revealed that the absolute time that animals spent in the sucrose zone in the condition *Juvenile vs. 10%* (*M* = 408, *SD* = 19) was significantly more than the condition *Juvenile vs. 5%* (*M* = 205, *SD* = 20, *p* < 0.001, *d* = 10.4) and the condition *Juvenile vs. 2%* (*M* = 159, *SD* = 15, *p* < 0.001, *d* = 14.5). No significant difference was found between the conditions *Juvenile vs. 2%* and *Juvenile vs. 5%*. As a follow-up analysis, a paired sample *t*-test per condition revealed that in Juvenile vs. 2%, the Juvenile side (*M* = 259, *SD* = 64) was significantly (*p* < 0.05, *d* = 1.7) preferred over the sucrose side (*M* = 159, *SD* = 53). In the condition *Juvenile vs. 5%*, animals were indifferent between the reward types (Juvenile: *M* = 250, *SD* = 76; 5% sucrose: *M* = 205, *SD* = 71). In contrast, in the condition *Juvenile vs. 10%*, the sucrose side (*M* = 408, *SD* = 67) was preferred significantly (*p* < 0.001, *d* = 5.6) over the Juvenile (*M* = 97, *SD* = 55) (see Figure 2E).

### Characterization of USV

As indicated in “Materials and Methods” section, the 50 kHz USVs produced by experimental animals in the SSPT were labeled and further categorized into subtypes. Descriptive statistics were generated for each of the subtypes included in our analyses, along with within-condition and between-condition comparisons. We found that rats emitted vocalizations in a total of *N* = 7,252 call frames (290 s, combined SDT, and SSPT, 2.4% of total recorded frames, Figure 3A). After exclusion of 22 kHz calls, based on prevalence, we selected eight subtypes: Trill (Tr), Flat (Fl), Complex (Cx), Trill-with-Jump (Tj), Short (Sh), Flat-Trill-combination (Ft), Split (Sp), and Composite (Ce) for further analysis (Figure 3F). Six subtypes (Step-Down, Step-Up, Upward Ramp, Multi-Step, Inverted-U, Downward Ramp) were excluded from analysis due to their limited occurrence (<2% of calling time, Figure 3D). From the selected subtypes, Tr (27.2%), Fl (24.4%), Cx (11.5%), and Ce (11.3%) were the most prevalent, while Sh (5.5%), Ft (4%), Sp (3.4%), and Tj (2.2%) were least prevalent in both tasks (Figure 3D). Notably, we found Un calls (3% in the SDT task, Figure 3B) and (6% in the SSPT task, Figure 3C. For more details about Un calls, see section “Materials and Methods”).

**SDT.** In total, throughout the SDT, 2155 call frames were found in which the rats were vocalizing, and after exclusion of 22 kHz calls, from the eight selected subtypes, Fl (48%), Tr (13%), Cx (10%), Sp (6%), Sh (5%), and Ce (5%) were most prevalent while, Ft (2%), and Tj (0.06%), were least prevalent in SDTs’ conditions (Figure 3B). **SSPT.** In total, in the SSPT, 5097 call frames were found in which the rats were vocalizing, and after exclusion of 22 kHz calls, from these eight selected subtypes, Tr (33%), Fl (14%), Ce (14%), Cx (12%) were most prevalent while Sh (6%), Ft (5%), Tj (3%), and Sp (2%) were least prevalent (Figure 3C) in SSPTs’ conditions.

### Analysis of Total USVs

To determine if the number of frames that the rat vocalized was affected by sucrose concentration or type of rewards in the different conditions, we conducted a two-way repeated-measures ANOVA on the Combined vocalization score (CVS; the number of frames vocalized relative to the time spent in the visited zone, see section “Materials and Methods”) with condition and reward zone as factors, separate for SDT and SSPT.

**FIGURE 4:**
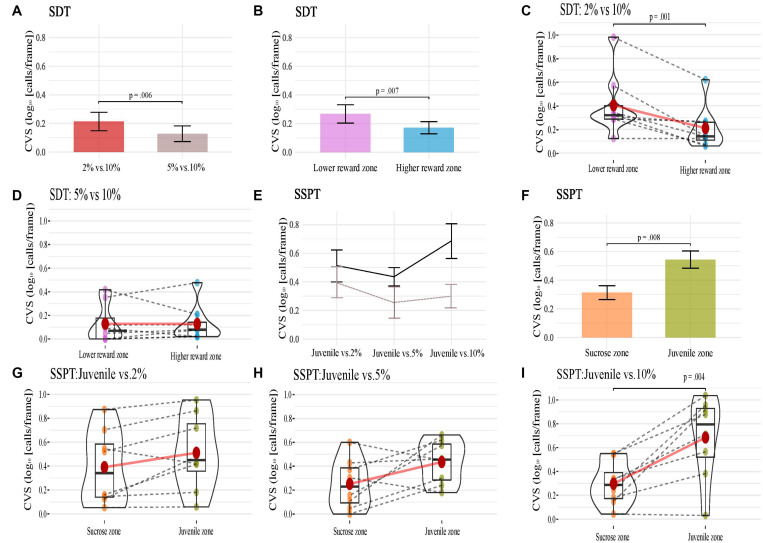
**(A–D)** Present animals’ CVS in conditions **(A)**, reward zones **(B)** of the SDT, and **(C,D)** show each animal’s CVS in the two reward zones of the SDT task’s two conditions. **(E–I)** show animals’ CVS across three conditions (**E**; in both reward zones, black solid line; Juvenile and brown hashed line; non-social), reward zones **(F)** of the SSPT and **(G–I)** show each animal’s CVS in the two reward zones of the SSPT task’s three conditions. Error bars indicate Standard Error for **(A,B,E,F)**.

**SDT.** The SDT analyses found a significant effect of condition on the CVS [*F*(1, 7) = 14.9, *p* < 0.01, η*_*p*_*^2^ = 0.680]. The main effect showed that the CVS was significantly higher in the condition *2% vs. 10%* (*M* = 0.310, *SE* = 0.075) than in the condition *5% vs. 10%* (*M* = 0.128, *SE* = 0.054; Figure 4A). The factor reward zone also had a significant effect on the CVS [*F*(1, 7) = 14.3, *p* < 0.01, η*_*p*_*^2^ = 0.672; Figure 4B]. The main effect showed that the CVS was, surprisingly, higher (*p* < 0.01) in the lower sucrose concentration zone (*M* = 0.268, *SE* = 0.065) compared to the higher sucrose concentration zone (*M* = 0.171, *SE* = 0.058). There was also a significant interaction effect of conditions and reward zones [*F*(1, 7) = 5.9, *p* < 0.05, η*_*p*_*^2^ = 0.459; Figures 4C,D). *Post hoc* comparisons showed that CVS was higher in lower-reward zones only for the condition *2% vs. 10%.* In the zone of lower sucrose concentration (*M* = 0.407, *SE* = 0.093) the animals had a higher CVS (*p* < 0.05) than the condition *5% vs. 10%* (*M* = 0.213, *SE* = 0.064, see Figures 4A,B). ***SSPT.*** For the SSPT task, we again performed a two-way within-subjects repeated-measures ANOVA. There was no significant effect of condition (Figure 4E), but we found a significant effect of reward type [*F*(1, 7) = 13.6, *p* < 0.01, η*_*p*_*^2^ = 0.658, Figure 4F]. *Post hoc* comparisons showed that the CVS was significantly higher in the Juvenile zone (*M* = 0.544, *SE* = 0.075) than in the sucrose zone (*M* = 0.313, *SE* = 0.067; *p* < 0.01). Furthermore, there was a significant interaction between condition and reward types [*F*(2, 14) = 5.1, *p* < 0.05, η*_*p*_*^2^ = 0.426, Figures 4E–I]. *Post hoc* comparisons showed that animals’ CVS in the Juvenile vs. 10% condition was significantly higher (*p* < 0.01) in the Juvenile zone (*M* = 0.685, *SE* = 0.121) compared to the sucrose zone (*M* = 0.297, *SE* = 0.064). No significant differences in CVS between reward zones were found for the Juvenile vs. 2% (*p* = 0.06) and Juvenile vs. 5% conditions (*p* = 0.07).

These results already indicate an interesting finding: while behavioral preferences shifted toward the sucrose reward zone with higher sucrose concentration, the vocalization rate showed the opposite trend, with increasing vocalizations recorded in the juvenile zone with increasing sucrose concentrations. We next investigated whether this pattern was present for specific subtypes and if there were differences between subtypes.

Comparing USV subtypes between and within conditions.

### Between-Subtypes Analyses

As one of the main questions of this study, we were interested in finding out if the different sucrose concentrations or different reward types were associated with a different vocalization palette across the 50 kHz USV subtypes. Here, we used the delta Subtype Vocalization Score (dSVS; see section “Materials and Methods”), indexing the relative difference in vocalization rates between reward zones in a given session for these analyses, as it accounts for normalization of inter-individual differences in absolute call rates.

**FIGURE 5:**
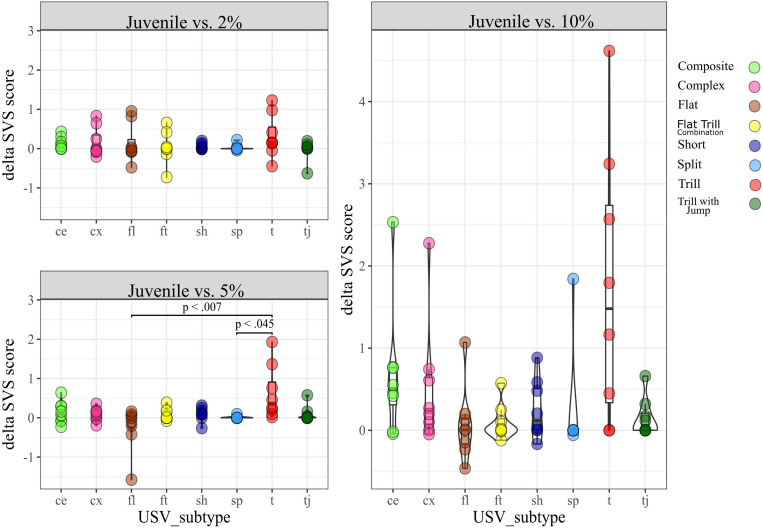
dSVS for each subtype, split by conditions in the SSPT.

**SDT**. We conducted a Kruskal Wallis test separately for each condition (*2% vs. 10%* and *5% vs. 10%*) by taking the eight subtypes observed in the SDT as a factor and their dSVS as the dependent variable (DV). We found no significant difference in the dSVS between subtypes for any condition (Supplementary Figure D).***SSPT.*** We similarly conducted a Kruskal Wallis test for each condition (*Juvenile vs. 2%*, *Juvenile vs. 5%*, and *Juvenile vs. 10%*). In the condition *Juvenile vs. 5%*, we found a significant difference [H (7) = 16.6, *p* < 0.05]. *Post hoc* pairwise comparisons showed a significant difference between dSVS of the subtypes Tr (median = 0.3) and Fl (median = −0.04), (Mann-Whitney U-test, *p* < 0.01) and dSVS of subtypes Tr and Sp (median = 0, *p* < 0.05; Figure 5).

### Within Subtype Analyses

**FIGURE 6:**
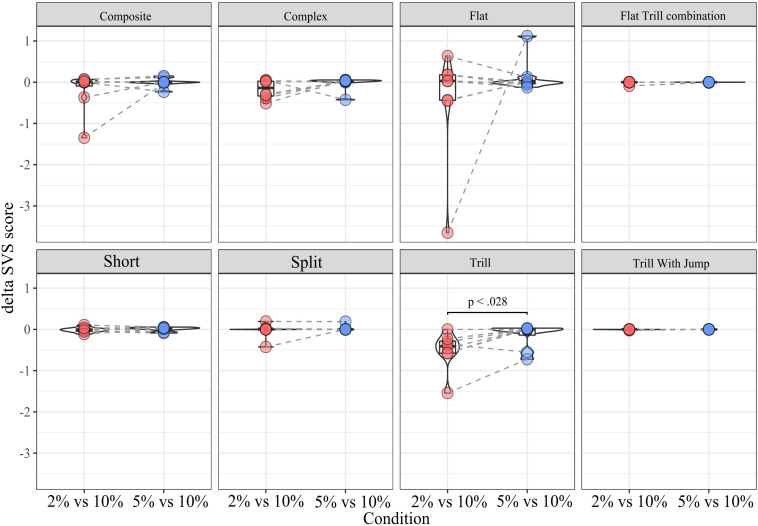
dSVS split by subtype between conditions.

***SDT.*** this analysis was conducted to determine whether dSVS for a given subtype differed between conditions. The Wilcoxon Signed-Rank test results showed that the dSVS score of Tr was lower in condition *2% vs. 10%* (median = −0.4) than in condition *5% vs. 10%* (median = 0), *Z* = 2.1, *p* < 0.05). There was no other significant difference within any subtypes between conditions (Figure 6).

### Mixed Linear Model Analyses

**FIGURE 7:**
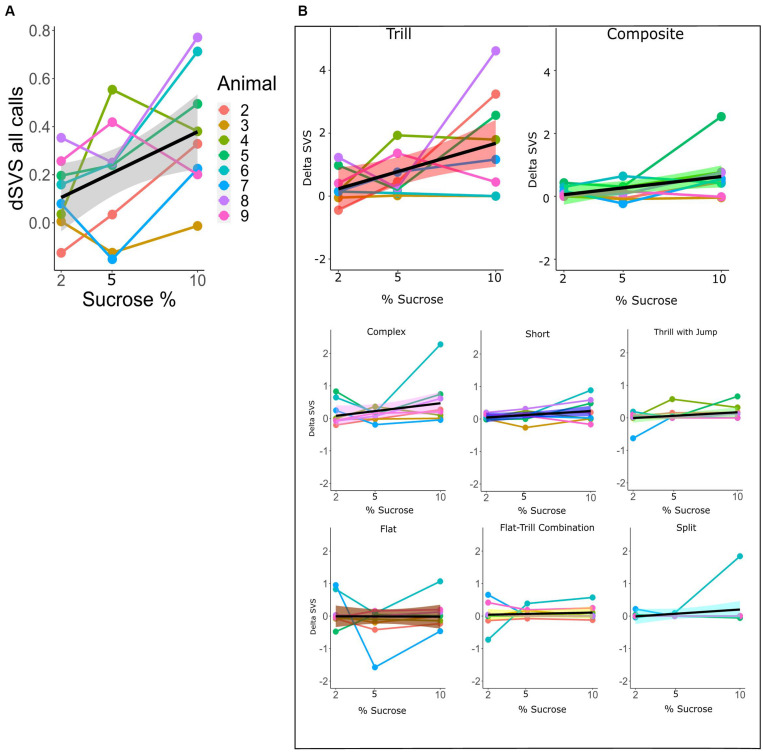
**(A)** dCVS for all calls across different levels of sucrose in the SSPT. The black line (±standard error of the mean; gray shade) shows the estimated linear relationship between dSVS and sucrose concentrations across all rats. Linked dots represent individual rats, modeled as a random effect. **(B)** Each plot shows the change in dSVS of a certain subtype across three SSPT task conditions. Black lines represent the mean linear trends across all rats and (±standard error of the mean is represented by shade; colored differently for each subtype.). The slopes for Trill and Composite subtypes are significant (Supplementary Table 2A).

For the within-subtype analysis of call rates in the SSPT, we exploited the continuous nature of the sucrose concentration in a mixed linear model, estimating the relationship between sucrose concentration (in %) and dSVS with individual animals modeled as random effects. We first modeled the total call rate (all calls combined) using the Combined vocalization score (delta CVS; see section “Materials and Methods”). The mixed linear model showed a linear association between the delta CVS and the sucrose level (beta = 0.034, 95% CI [0.01–0.06], *t*(15) = 3.27, *p* < 0.01, R2 fixed effect = 0.208). This suggests that the difference in total vocalization time in the Juvenile over the Sucrose zone significantly *increased* with higher levels of sucrose concentration (see Figure 7A and Supplementary Table 1). We then modeled the sucrose concentration to delta SVS relationship in linear mixed models separately for each subtype. The models showed a significant association for the subtypes Tr (beta = 0.18, 95% CI [0.05–0.031], *p* < 0.05) and Ce (beta = 0.07, 95% CI [0.01–0.013], *p* < 0.05). This means that, for these two subtypes, the difference in the number of frames vocalized in the Juvenile over the Sucrose zone significantly increased with higher levels of sucrose concentrations (see Figure 7B and Supplementary Tables 2A,B for more individual model statistics).

## Discussion

Communication is essential for social animals, and rats are no exception. Rats utilize vocalizations in the ultrasonic range to communicate with their conspecifics. However, whether these vocalizations differ in response to different rewards when presented together and whether vocalizations quantitatively index reward magnitude remained mostly unexplored.

Here, we presented a paradigm to test preferences for two different reward types head-to-head in distinct spatial locations on a four arm-maze. We simultaneously quantified social vs. non-social reward value through relative reward zone time allocation and reward type preference profiles by estimating slopes over three clearly discriminable (Figure 2C) non-social reward values (sucrose concentrations). Rats, indeed, changed their time allocation over reward sites as a function of reward sucrose concentration (Figure 2E) and even exhibited preference reversals, switching from preferring social interaction when it competed with 2% sucrose to preferring sucrose consumption when its concentration was upped to 10%. This change in behavioral preference and time allocation could be exploited to estimate the association between different 50 kHz USV subtypes and social vs. non-social reward, controlling for individual differences in overall vocalization rate and variance in time spent at each reward site (Figure 4).

We found that, when controlling for occupancy and individual differences in this way, the overall difference in vocalization rate between social and non-social reward sites (dCVS; normalized vocalization rate social minus non-social) increased from 2 to 5 to 10% sucrose conditions, as estimated with a linear model, suggesting that animals vocalized *more* in the social zone even though the experimental animals spent *less* time in the social side when the alternative was a high-sucrose solution. The vocalization rate was not purely determined by appetitive sucrose consumption either, as witnessed by the dramatic reduction in call rate in the SDT conditions, even though animals exhibited comparable levels of sucrose consumption and behavioral preferences. As several studies already showed, 50 kHz USV calls are emitted during various appetitive states (Brudzynski and Zeskind, 2018), such as sucrose consumption and social play (Browning et al., 2011). Therefore, we hypothesized that, in the SDT task, more calls would be emitted in the *5% vs. 10%* condition than the *2% vs. 10%* condition (overall more sucrose) and that a higher percentage of calls would be scored in the higher sucrose zone in both conditions. Both hypotheses were *rejected*, however, as the rats vocalized significantly *more* in the *2% vs. 10%* condition, controlling for occupancy and more calls we found in the *lower sucrose* zone in both conditions.

These findings, thus, rather support a view of USVs as a context-dependent communicative device aimed perhaps at establishing/inviting social contact compared to the alternative hypothesis that casts USVs as (static) epiphenomena of reward value linked to the consumption of social contact or non-social rewards. Many researchers have pointed to the associations between the various 50 kHz USV subtypes and certain types of overt behavior (Wöhr et al., 2008; Wright et al., 2010; Mulvihill and Brudzynski,2018a,b). When we zoomed in to the level of the various 50 kHz subtypes, we found that in our experiments, eight subtypes (Tr, Fl, Cx, Tj, Sh, Ft, Sp, and Ce) were vocalized much more prevalently than the other remaining subtypes identified by Wright et al. (Wright et al., 2010). We thus investigated whether the vocalization rate of these subtypes could be used to discriminate between Social and non-social reward-related contexts.

When considering the SDT sessions, the Flat subtype was vocalized at a much higher rate compared to the remaining eight selected subtypes (Figure 3B). This parallels the findings of Mulvihill and Brudzynski (2018b), who reported that non-social conditions appeared to induce a greater proportion of flat calls as well as the findings of Wöhr and Schwarting (2013), who found an association of flat 50 kHz USVs and feeding behavior. Likewise, Wright et al. (2010) also found that flat calls were more prevalent in singly-tested rats than pair-tested rats. However, in our hands, the proportion of flat calls across high- and low-reward zones (dSVS) did not differ between flat calls and the other subtypes (Supplementary Figure D) or across SDT conditions for flat calls (Figure 6), arguing against a direct, parametric association between flat calls and hedonic state.

In contrast, similar to the findings of Brudzynski and Pniak (2002) and Wright et al. (2010), demonstrating that animals generally vocalize more in the presence of conspecifics, in the SSPT, our subjects also vocalized more in the social reward zone than the non-social reward zone. Moreover, sucrose levels influenced this effect as conditions with a competing higher concentration of sucrose elicited higher vocalization of 50 kHz USVs in the social zone (Figure 7A). This result parallels the results of Mulvihill and Brudzynski (2018a), who demonstrate that social contexts in particular conditions induce call emission more robustly. In particular, the Trill and Composite subtypes drove this effect and were produced at increasing rates in the social zone when animals were deciding between visiting the Juvenile and increasing sucrose (Figure 7B). This finding becomes particularly interesting when considering that animals spent more time at the *non-social* zone at higher sucrose concentration conditions (see; Figures 2E, 4E). What could explain this inverse relationship between behavioral preferences and differential USV production? We offer three putative explanations:

(1) The sessions with higher sucrose concentrations induce an overall higher hedonic state that potentiates “chattiness” when the experimental animal visits the Juvenile zone.
(2) The higher sucrose content influences the breath of the experimental animal, which in turn modulates the USV production when the animals are interacting.
(3) With increasing sucrose concentration, the experimental animal shuttle more and faster between reward sites (anecdotal observations). If USV production decays exponentially with interaction time, shorter interactions yield a higher (normalized) call rate.

## Limitations and Future Directions

Adjudicating between these options will require further studies. One important limitation worth mentioning is that we utilized rats raised and tested in laboratory conditions. In a sense, our design is a drastically simplified version of what a rat might encounter in naturalistic settings. Studies such as ours aimed at elucidating the intricate patterns and subtypes of vocalizations in a micro-scale should be consolidated with field studies and naturalistic designs of rodent vocal behavior. Another important limitation of our study is that when the experimental animal was in the juvenile arm, we were unable to determine precisely whether the experimental or juvenile animal was vocalizing. Though several attempts have been made, using triangulation, microphone arrays (Heckman et al., 2017), or onboard wireless EMG recordings of the larynx (Kelm-Nelson et al., 2018) to arrive at precise disambiguation of the USV source, the current setup did not allow this objective to be met in our study. Previous research has shown that, in juvenile rats, a positive correlation between the emission of 50 kHz USV vocalizations and rough-and-tumble play could be found (Knutson et al., 1998; Kisko et al., 2015), and that devocalization in the pair impacts social play (Himmler et al., 2014). In our design, most (but not all) rats increased their total vocalization from SDT to SSPT task (Figure 3E). Though we attribute this increase mostly to the addition of the juvenile, we still observed vocalizations with the strongest amplitude on the microphone over the non-social side (data not shown), presumably originating from the experimental animal, arguing against the vocalization originating only from the juveniles. Considering the findings of Wöhr and Schwarting (2007, 2012) that 50-kHz USV constantly gave rise to social approach behavior in juvenile and adult male rats, we interpret our finding of more USVs emitted per second spend investigating the juvenile as a corollary of the juvenile inviting social contact through vocalizations, growing stronger as the experimental animal is spending more time in the non-social zone with increasing sucrose concentration.

Taken together, our study provides a first systematic overview of behavioral preferences and vocalization patterns recorded when rats are choosing between social and non-social rewards. The underlying behavioral and/or genetic traits and the neural correlations regulating the rats’ specific preferences are yet to be explored. Recent studies utilizing a combination of cutting edge genetic techniques to pinpoint neural underpinnings of rodent vocal communication (Kisko et al., 2018; Gao et al., 2019; Tschida et al., 2019) have illustrated the value of rodent models in elucidating the social behavior and pro-social 50-kHz ultrasonic communication as models of psychiatric illness. Our results again highlight the variance in rat vocalizations between individuals and within their repertoire. Not only did the total number of USVs differ depending on the type of and level of reward, but the specific subtypes themselves showed variation between conditions and rewards, and in some cases, were predictive of the level of reward. So what is the ultimate role of the different USV subtypes? We and others propose that these USV subtypes allow rats plasticity in their vocal behavior, enabling flexible communication to respond to the (social) cues from their surroundings appropriately. The conditional probability of one subtype following another is not random (Coffey et al., 2019), suggesting the possibility of syntax, or perhaps even turn-taking in an interacting rodent dyad. Such analyses could be combined with data-driven approaches to USV categorization that include frequency and/or amplitude information and machine learning in addition to expert-based pattern recognition of USV subtypes. Creating synthetic USV sequences that could outperform random sequences in eliciting approach behavior, now used as the gold standard (Seffer et al., 2014), would indicate the importance of subtypes in a USV call structure.

## Data Availability Statement

The original contributions presented in the study are included in the article/Supplementary Material, further inquiries can be directed to the corresponding author/s.

## Ethics Statement

The animal study was reviewed and approved by the European Union Directive 2010/63/EU for animal experimentation and was approved by the local authority (Landesamt für Natur, Umwelt und Verbraucherschutz North-Rhine Westphalia, Germany).

## Author Contributions

MW and MS contributed to the conception and design of the study. MS executed the study, collect the data, did statistical analyses, created the figures, and wrote the manuscript. SG, NP, and IC contributed to writing R script, creating graphs, figures, and tables, and also writing the introduction and discussion. TK and MW contributed to manuscript revision. All authors approved the submitted version.

## Conflict of Interest

The authors declare that the research was conducted in the absence of any commercial or financial relationships that could be construed as a potential conflict of interest.
