# Supplementary figures and images for: Distinct Profiles of 50 kHz Vocalizations Differentiate Between Social Versus Non-social Reward Approach and Consumption
Source: Front Behav Neurosci. 2021 Jun 21;15:693698. doi: 10.3389/fnbeh.2021.693698 (PMC8255485; doi:10.3389/fnbeh.2021.693698)

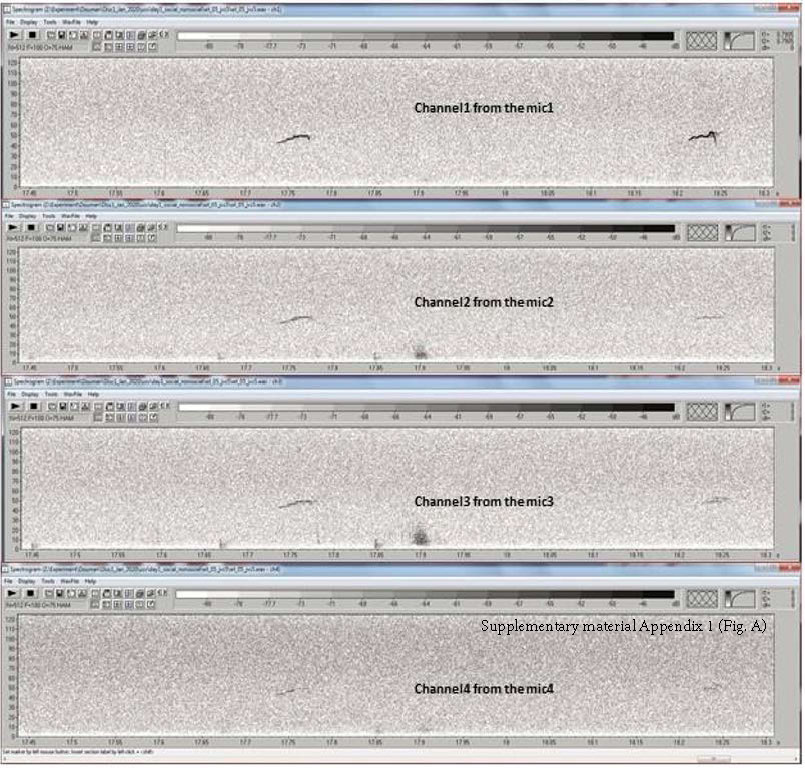

Supplement: Supplementary file 1 [file Image_1.JPEG]

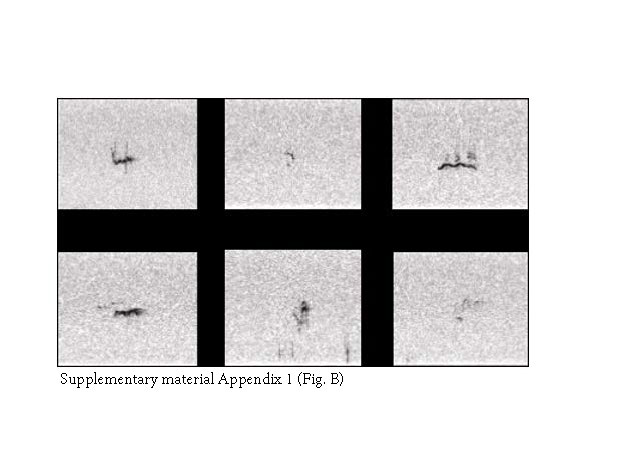

Supplement: Supplementary file 2 [file Image_2.JPEG]

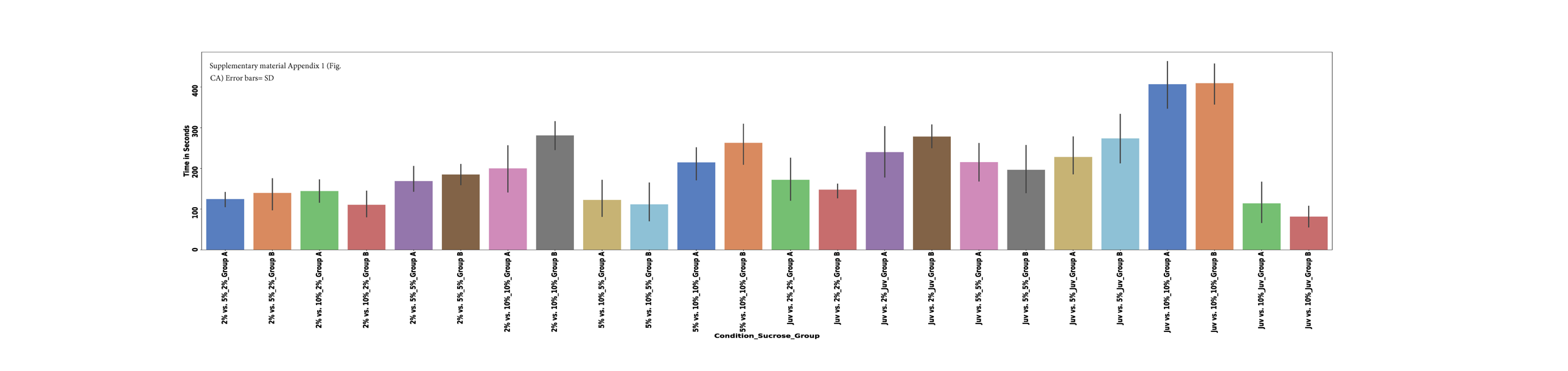

Supplement: Supplementary file 3 [file Image_3.JPEG]

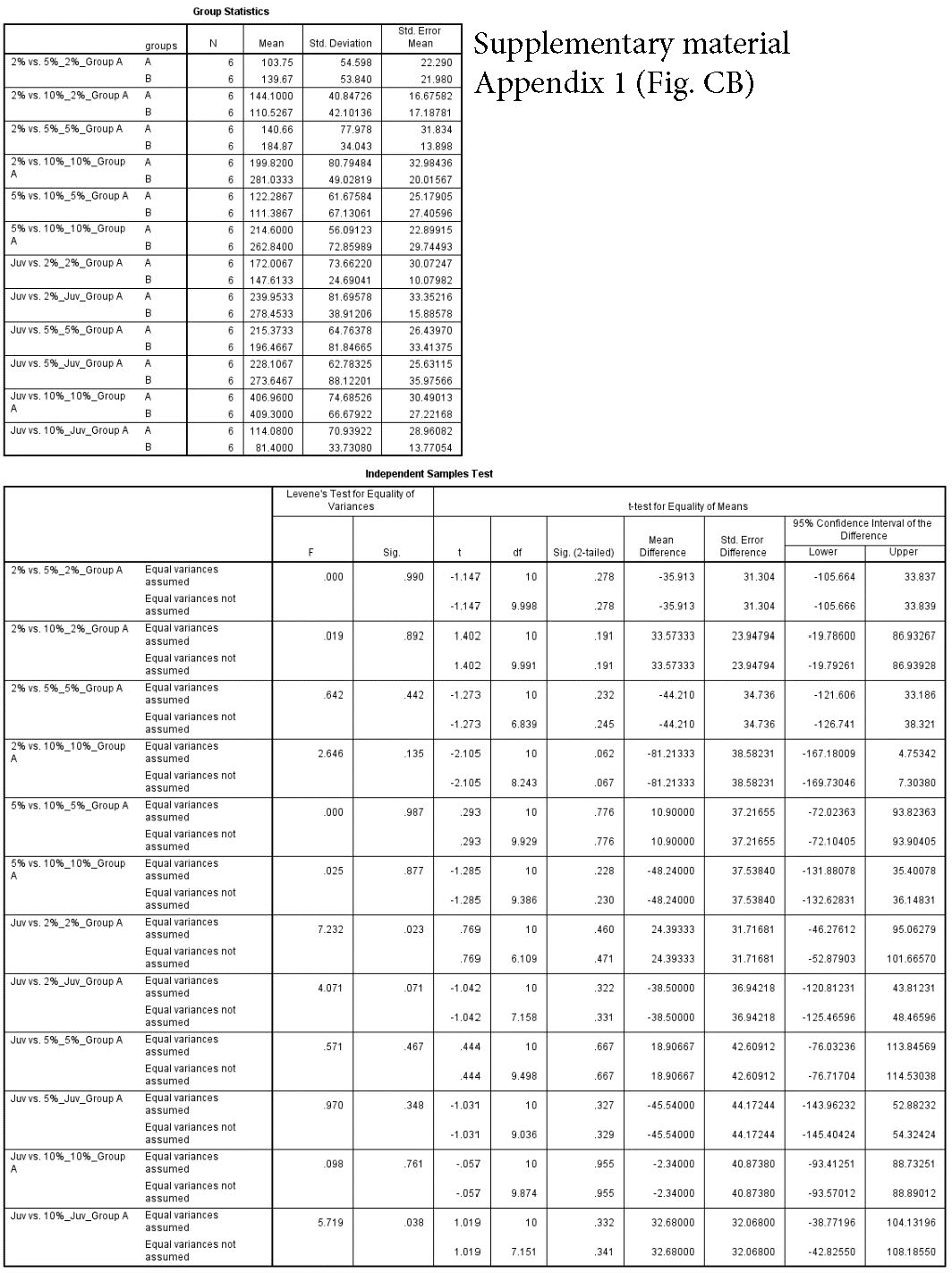

Supplement: Supplementary file 4 [file Image_4.JPEG]

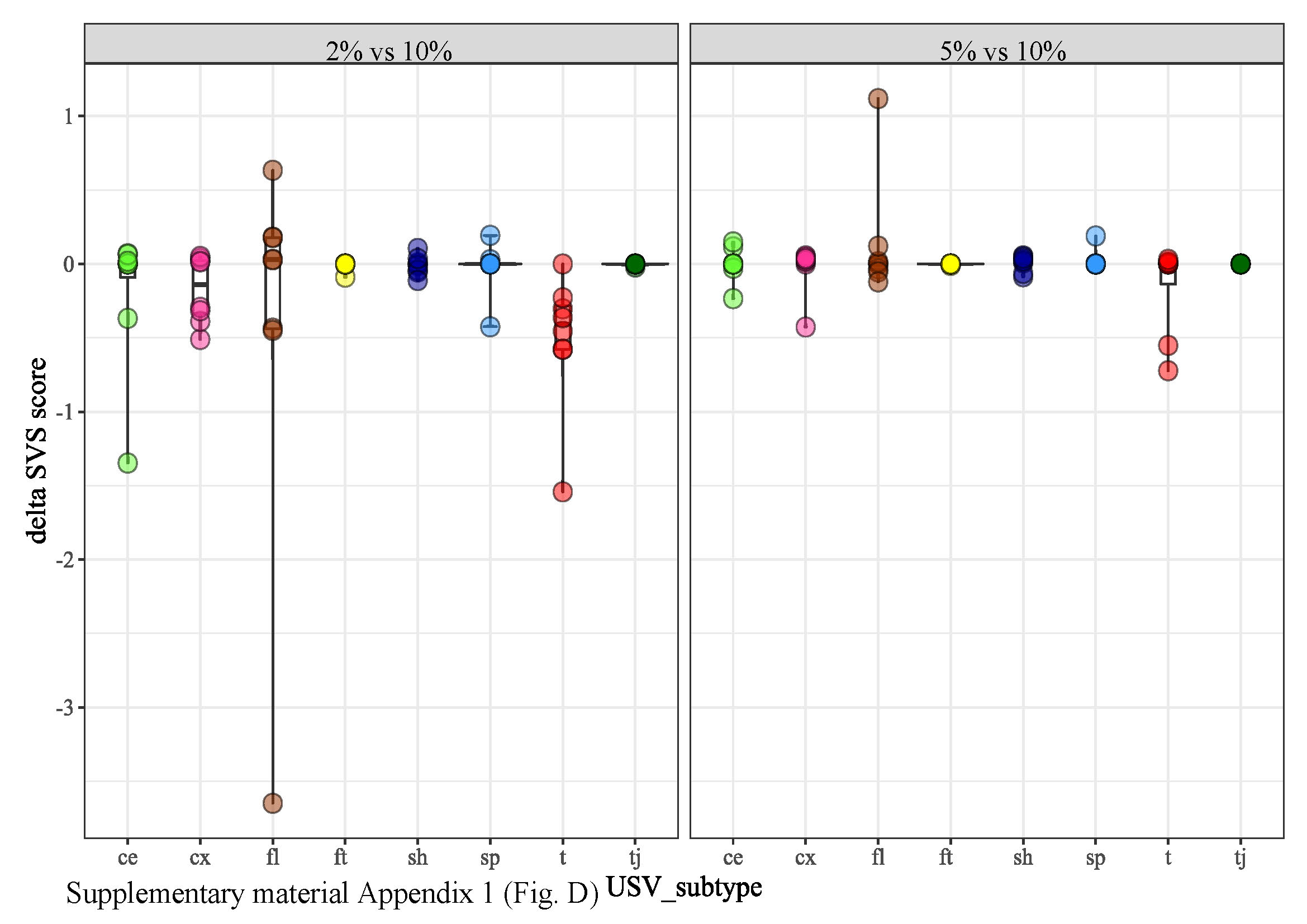

Supplement: Supplementary file 5 [file Image_5.JPEG]

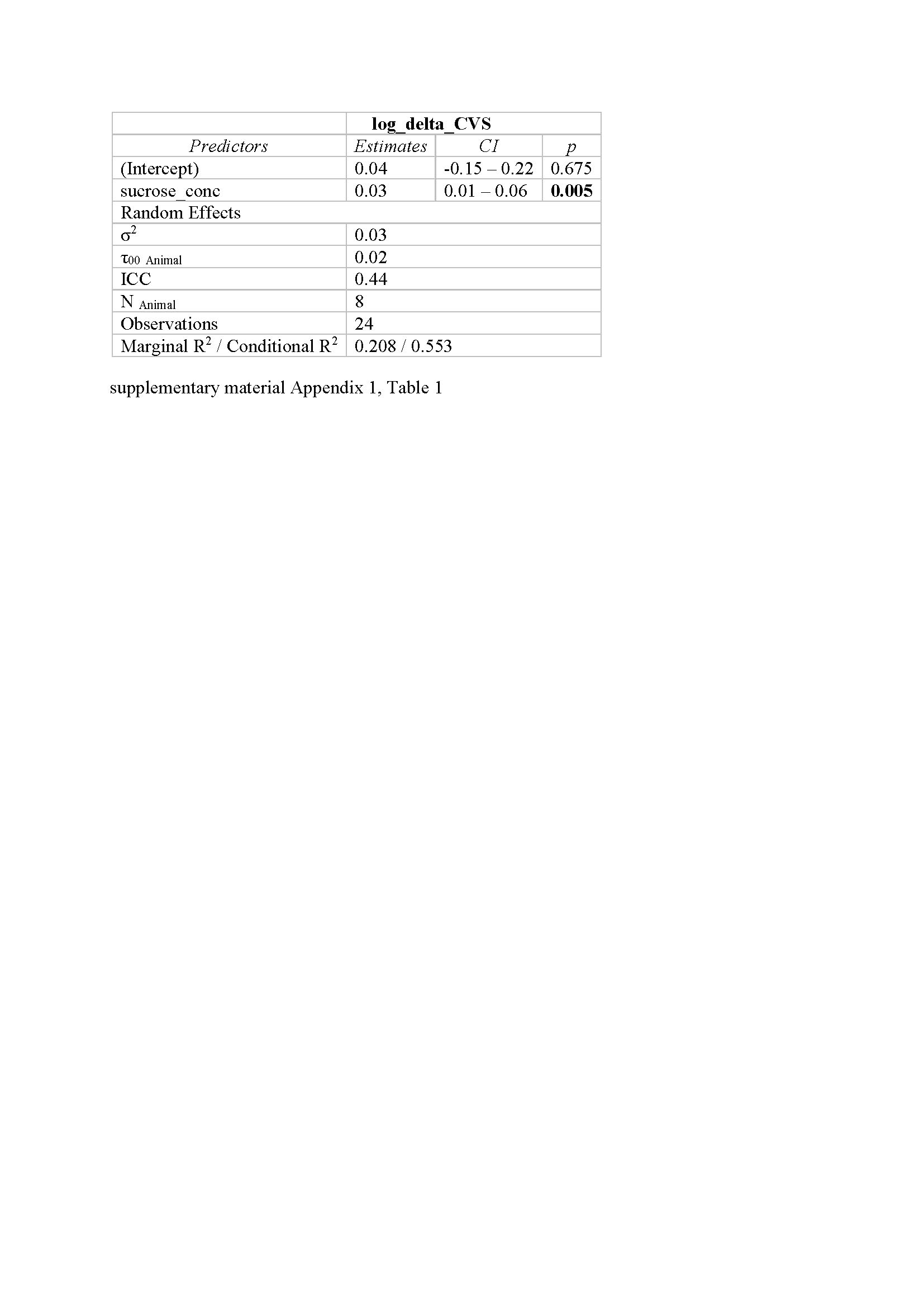

Supplement: Supplementary file 6 [file Image_6.JPEG]

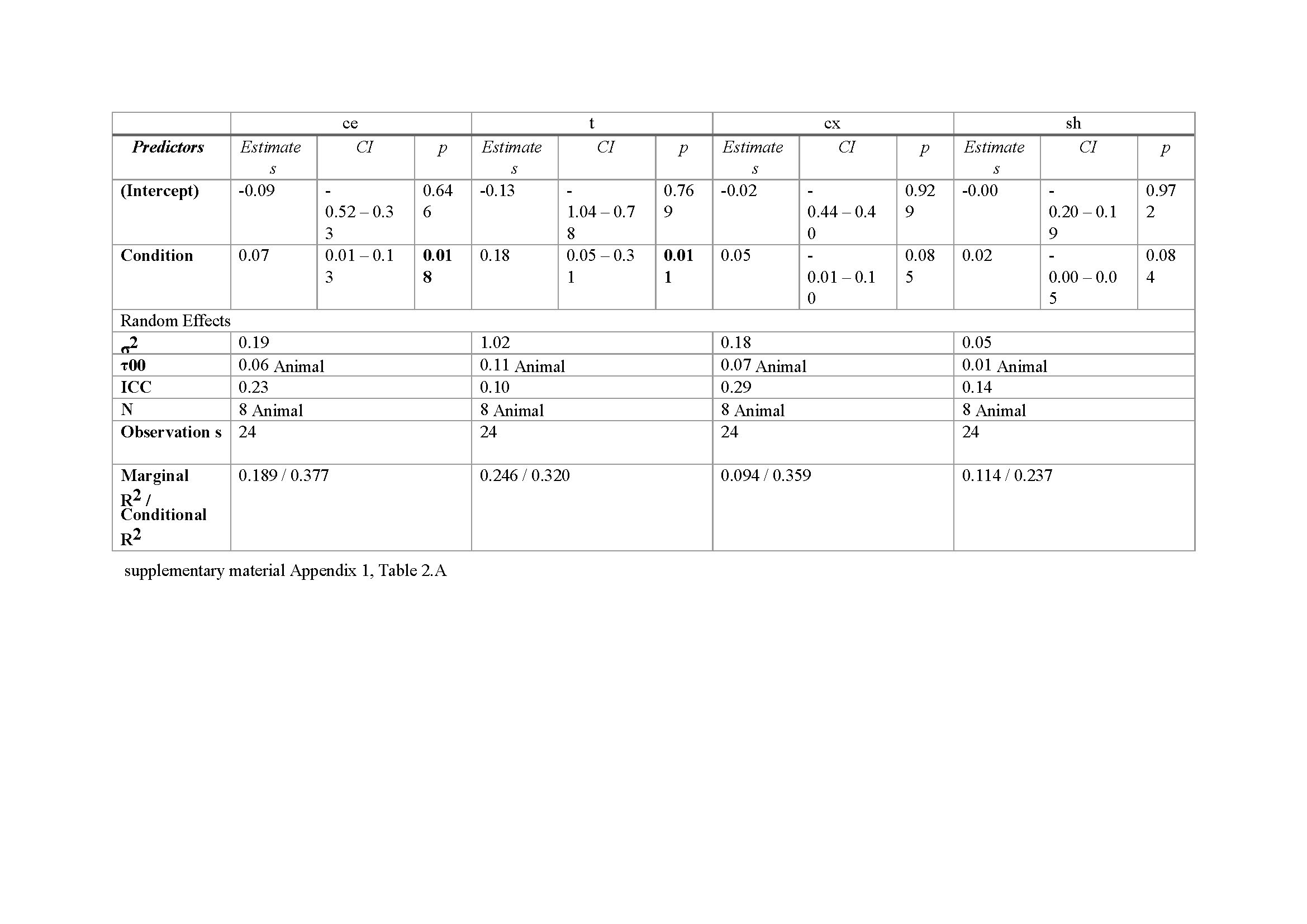

Supplement: Supplementary file 7 [file Image_7.JPEG]

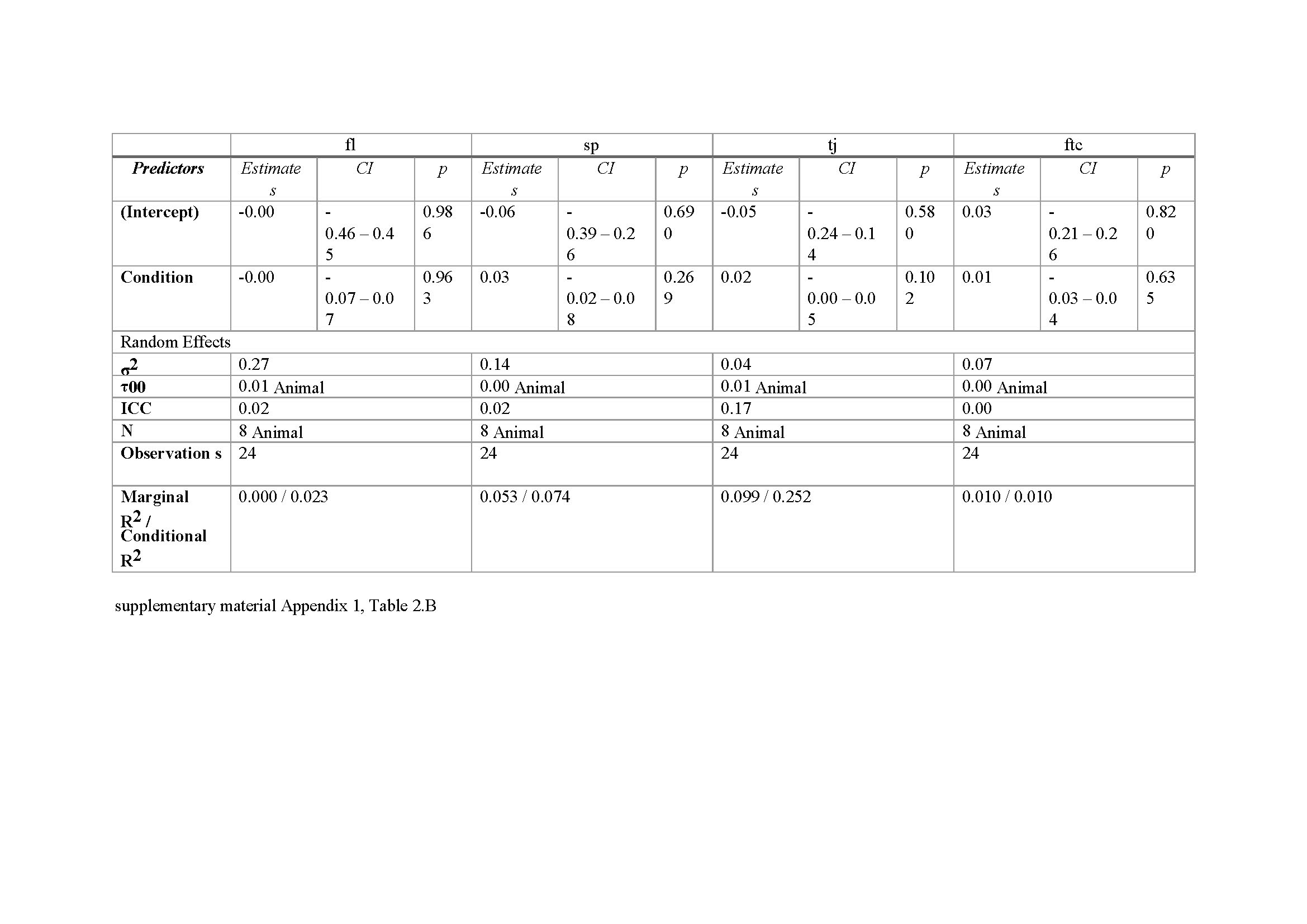

Supplement: Supplementary file 8 [file Image_8.JPEG]
